# TRPV4 induces apoptosis via p38 MAPK in human lung cancer cells

**DOI:** 10.1590/1414-431X2021e10867

**Published:** 2021-10-18

**Authors:** Yanyan Zhao, Jiaying Wang, Xuehui Liu

**Affiliations:** 1 Department of Respiratory Medicine, Second Hospital of Shanxi Medical University, Taiyuan, Shanxi, China

**Keywords:** Lung cancer, TRPV4, Apoptosis, P38 MAPK, Proliferation

## Abstract

Lung cancer is one of the most common cancers worldwide. TRPV4 belongs to the ‘transient receptor potential' (TRP) superfamily. It has been identified to profoundly affect a variety of physiological processes, including nociception, heat sensation, and inflammation. Unlike other TRP superfamily channels, its roles in cancers are unknown. Here, we elucidated the effects of TRPV4 and molecular mechanisms in human lung cancer cells. The levels of TRPV4 were detected in human lung cancer tissues and the paired paracarcinoma tissues by real-time PCR and western blotting analysis. The proliferation of human lung cancer cells was determined by MTT assay. Cell apoptosis was determined by FACS assay. The results demonstrated that low levels of TRPV4 were detected in clinical lung carcinoma specimens. Over-expression of TRPV4 induced cell death and inhibited cell proliferation and migration in A549 cells and H460 cells. Moreover, over-expression of TRPV4 enhanced the activation of p38 MAPK signal pathway. Inhibition of p38 MAPK abolished the effects of TRPV4 on cell proliferation, apoptosis, and migration in A549 cells. Collectively, our findings indicated that TRPV4 induced apoptosis via p38 MAPK in human lung cancer cells and suggested that TRPV4 was a potential target for therapy of human lung cancers.

## Introduction

Lung cancer (LC), also called lung carcinoma, is a malignancy characterized by elevated morbidity as well as mortality not only in China, but also worldwide (1). LC is mainly classified as either small cell carcinoma (SCLC) or non-small cell lung cancer (NSCLC) (2,3). Frequently seen types of NSCLC include large cell carcinoma, squamous cell carcinoma, and adenocarcinoma (4). Several risk factors have been identified for the initiation of LC, including cigarette smoking, exposure to radon, chromates, air pollution, second-hand smoke, arsenic, nickel, and radiation therapy (5,6). Despite significant progress in treating LC, the clinical outcome and patient survival is far from satisfactory. This may be attributed to a lack of early diagnosis and treatment strategies, as well as to complications related to the malignancy (7,8). Consequently, investigations of the mechanisms of LC generation as well as innovative strategies for the early diagnosis and treatment of LC are of great clinical importance.

Stimulation of programmed cell death, including the apoptosis of LC cells, may have essential implications for treatment (9). Transient receptor potential channel subtype 4 (TRPV4) is a polymodally modulated channel with appreciable Ca^2+^-permeability, that is able to transduce various physicochemical stimuli into calcium signals inside the cells (10-12). TRPV4 inhibition can prevent and resolve pulmonary edema due to heart failure (13). Experiments with GSK1016790A, a TRPV4 activator, led to endothelial/epithelial barrier disturbance, circulatory collapse, as well as lung edema in murine models (14). Moreover, it has been proven that TRPV4 reinforces malignancy extravasation in breast cancer and serves as a predictor of poor clinical outcomes in some solid epithelial malignancies (15,16). Furthermore, it has been shown that endothelial TRPV4 modulates vessel generation in tumors (17). Pharmacological TRPV4 stimulation in human melanoma cells and keratinocytes led to necrosis, cell death, and cellular disarrangement (18). Nevertheless, knowledge of the effect of TRPV4 on LC is still insufficient. We, therefore, investigated the effect of TRPV4 on human LC tissues as well as its molecular mechanisms on the progression and generation of LC.

## Material and Methods

### Patients and tissue samples

Our research was carried out from May 2016 to May 2017. Fifteen patients (nine men and six women) with an accurate pathology diagnosis of NSCLC were enrolled in this study. Lung tissues were acquired via surgery. None of the patients had undergone chemotherapy or radiotherapy. Fresh samples were immediately frozen in liquid N_2_ and preserved at −80°C, until used for the evaluation of TRPV4 expression via real-time PCR (RT-PCR) and western blot. LC tissues as well as matched surrounding normal samples were acquired from patients who provided informed consent at the Provincial Hospital before the research began. The study protocol was approved by the Ethics Committee of Second Hospital of Shanxi Medical University.

### Cell lines

Human lung adenocarcinoma cell line A549 (Cat. number TcHu150) and large cell LC cell line H460 (Cat. number TcHu205) were purchased from the Cell Resource Center of the Shanghai Institutes for Biological Sciences, Chinese Academy of Sciences. LC cells were cultured in DMEM medium including 10% fetal bovine serum (FBS), 1% streptomycin, and 1% penicillin.

### RNA isolation and quantitative RT-PCR

LC samples and matched non-malignant tissues surrounding the tumors were processed as follows. The isolation of total RNA of tissues was performed using TRIzol (Life Technologies, USA), and RNA was subsequently purified with the RNeasy Mini kit (Qiagen, Germany) according to the manufacturer's protocol. Superscript III kit (Life Technologies) was used for reverse transcription of complementary DNAs, which were then quantified via quantitative RT-PCR. The relative expression of mRNAs was measured via RT-PCR using SYBR Green PCR Supermix kit (Bio-Rad Laboratories, USA). Primers used were: TRPV4 forward primer, 5′-TTTGCTCTTATTTCTACTCCTCCC-3′; TRPV4 reverse primer, 5′-GCTGGCTTAGGTGACTCC-3′; GAPDH forward primer, 5′-CCACCCATGGCAAATTCCATGGCA-3′; GAPDH reverse primer, 5′-TCTAGACGGCAGGTCAGGTCCACC-3′. For every specimen, reactions were carried out in triplicate for no less than three independent runs. Expression data were analyzed with RealTime StatMiner® (Integromics, Spain). GADPH served as an internal control. Fold change was determined via relative quantification (2^-△△Ct^).

### Cell transfection

TRPV4-pcDNA3.1 (TRPV4) as well as pcDNA3.1 (Con) plasmids were used for cell transfection. In short, H460 and A549 cells at a density of 4×10^5^ cells/well were incubated in six-well plates for 24 h. Four micrograms of plasmid DNA, as well as three microliters of Turbofect reagent, were then added to the cultivating media followed by another 6 h of incubation. The transfection admixture was then removed and the cells were incubated for another 24 h in normal media.

### Cell survival assays

The MTT (3-(4, 5-dimethyl-2-thiazolyl)-2, 5-diphenyl-2-tetrazolium bromide) assay was used to measure the survival of the cells 48 h post-transfection. The cells (5×10^4^ cells/mL) were inoculated onto 96-well plates, and cultivated at 37°C in 5% CO_2_. MTT (0.5 mg/mL) was added into the culture medium for 4 h. Then, the MTT solution was aspirated and dimethyl sulfoxide (200 μL/well) was added. Absorbance of the supernatant was read at 490 nm using a microplate spectrophotometer (Thermo Scientific, USA). Absorbance was normalized to the untreated control cultures, which represented 100% viability. The formula %viability = mean absorbance of sample / mean absorbance of control ×100 was used. Experiments were performed in triplicate.

### Cell migration assays

The evaluation of cell migration was detected by transwell assays. At first, we suspended 5×10^4^ cells in DMEM without serum containing 1 μg/mL mitomycin C, and then added the mixture to wells on top of poly-carbonate transwell filters (Millipore, USA) in 24-well plates. Afterward, we added DMEM supplemented with 10% serum into the bottom of each well. The cells on the top of the filter were removed after 24 h of incubation, whereas those on the bottom underwent fixing, staining, and quantification.

### Assessment of cell death

We measured the apoptosis via flow cytometry. We washed the cells twice in cold PBS, centrifuged at 1000 *g* at 4°C for 5 min and discarded the supernatant. The pellet was resuspended in binding buffer, and propidium iodide with FITC-Annexin V were added to the mixture, followed by incubation for 10 min at room temperature. The mixture was finally processed through a FACScan flow cytometer (Guava Easy Cyte™8, Millipore, USA) in order to determine the cellular fluorescence.

### TUNEL assay for dead cells

Cells were fixed with 4% paraformaldehyde and the *in situ* Cell Death Detection kit (Roche, Switzerland) was used to conduct TUNEL labeling of dead cells according to the manufacturer's instructions. Fixed cells were incubated with bromodeoxyuridine triphosphate (Br‐dUTP) and terminal deoxyribonucleotidyl transferase (TdT), which bind the Br-dUTP to the 3′-hydroxyl end of the DNA fragment. The Br-dUTP was detected using FITC-labeled anti-BrdU monoclonal antibody and the DNA was counterstained with either propidium iodide (PI) or 4,6-diamidino-2-phenylindole dihydrochloride (DAPI) for image analysis. BSA (0.5%) was added to wash buffers to reduce cell loss. Parallel negative controls with distilled water instead of TdT were run for each sample.

### Western blots

Lysates as well as tissues were homogenized with lysis buffer (Beyotime, China) and protein was quantified by Bradford assay (Bio-Rad). Proteins were assessed with standard SDS-PAGE. Proteins were separated on 8-15% Tris-HCl polyacrylamide gels (Bio-Rad), before being transferred to a polyvinylidene difluoride (PVDF) membrane (Millipore). For incubation, the blots were placed in TBST overnight at 4°C in the presence of primary antibodies (anti-Bcl2, anti-caspase-3, anti-ERK, anti-JNK, anti-p38, anti-TRPV4, anti-Bax, anti-p-ERK, anti-p-JNK, anti-p-p38, and anti-β-actin) bought from Cell Signaling Technology (USA). They were subsequently incubated with secondary antibodies in conjugation with horseradish peroxidase. Enhanced chemiluminescence plus detection reagent (Pierce, USA) was used to visualize the immunoreactive bands, which were imaged via the Omega 16ic Chemiluminescence Imaging System (Ultra-Lum, USA).

### Statistical analysis

Results are reported as means±SE. Significant differences between groups were determined via two-tailed, unequal-variance Student's *t*-test, or ANOVA prior to Tukey's *post hoc* analysis. Results with P<0.05 were deemed significant.

## Results

### TRPV4 was down-regulated in LC specimens

**Figure 1:**
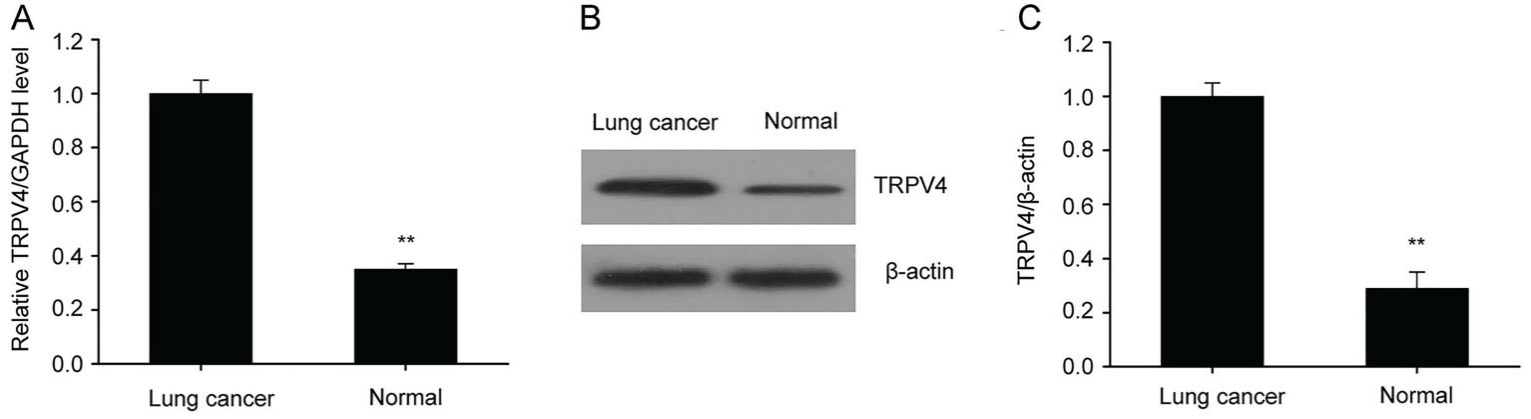
TRPV4 downregulation in lung cancer (LC) samples. **A**, qRT-PCR was carried out to evaluate TRPV4 expression in both LC tissues and surrounding non-malignant tissues (normal). **B** and **C**, Representative immunoblots and quantitative assessment of TRPV4 in human LC tissues and in surrounding non-malignant tissues (normal). Data are reported as means±SE (n=15). **P<0.01 (*t*-test).

To explore the importance of TRPV4 expression in LC, RT-PCR and western blots were carried out in 15 lung carcinoma tissue samples and matched non-malignant surrounding tissue samples. Relative TRPV4 transcription and translation levels were lower in LC than in the matched non-malignant tissues (Figure 1). These findings suggested that TRPV4 may serve to repress malignancy during generation as well as progression of LC.

### Overexpression of TRPV4 inhibited the proliferation of LC cell lines

**Figure 2:**
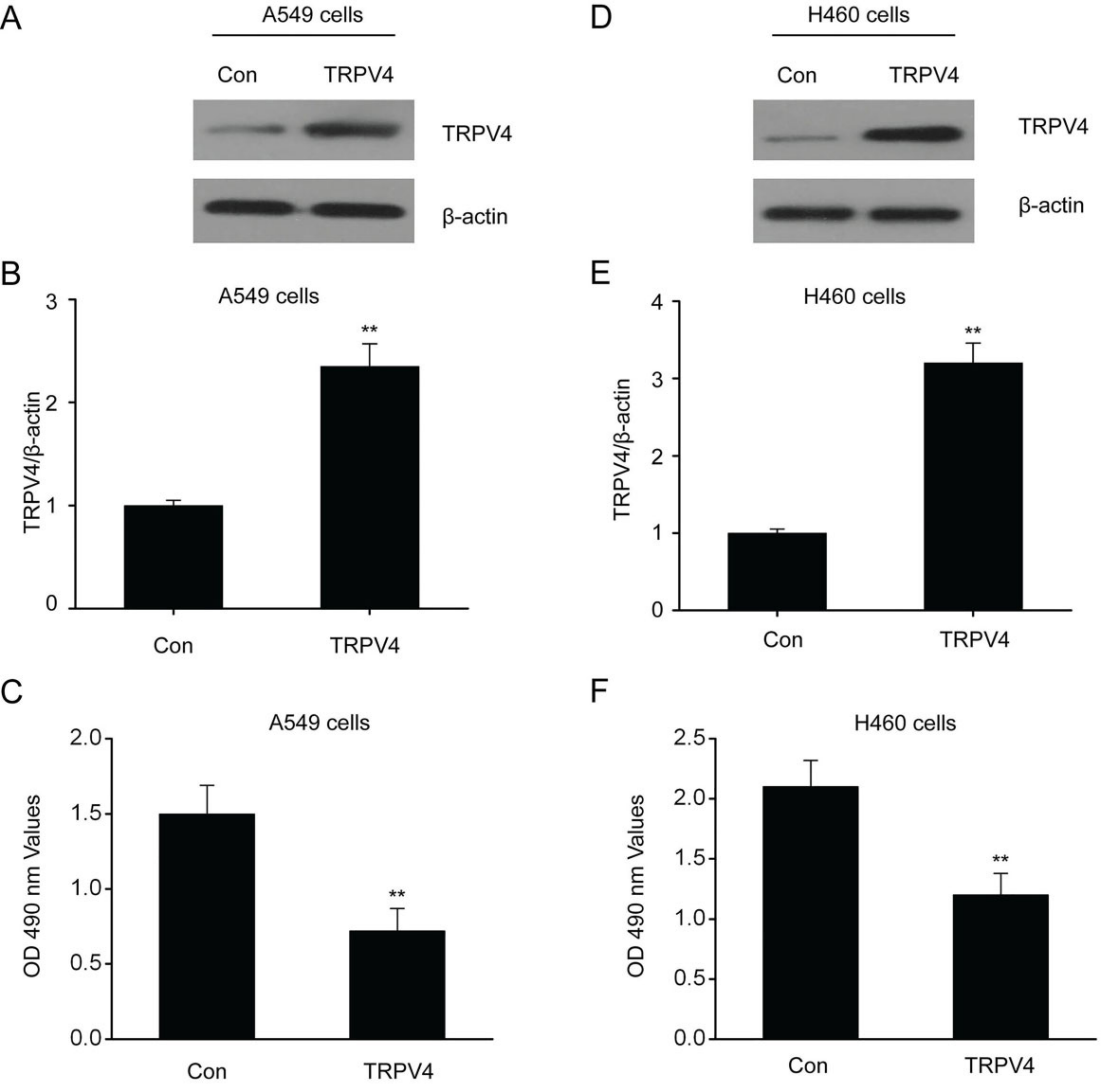
Overexpression of TRPV4 inhibited the proliferation of lung cancer (LC) cell lines. LC cells A549 (**A** and **B**) and H460 (**D** and **E**) were transfected for 24 h with the plasmid pcDNA3(+)/TRPV4 (TRPV4) or the control plasmid pcDNA3.1(+) (Con). Overexpression of TRPV4 was confirmed with western blot. TRPV4 inhibited proliferation as assessed via MTT assays in A549 (**C**) and H460 (**F**) cells. Data are reported as means±SE. **P<0.01 (*t*-test).

The LC cell lines H460 and A549 were transfected for 48 h with pcDNA3/TRPV4 or control plasmids. The TRPV4 concentration, as shown by the western blot, was remarkably elevated in the H460 and A549 cells transfected with the TRPV4 plasmids (Figure 2A, B, D, and E). Finally, an MTT assay performed to determine cell proliferation revealed that survival was notably lower in cells containing the TRPV4 plasmids than that in the controls (Figure 2C and F).

### Overexpression of TRPV4 expression inhibited migration of LC cell lines

**Figure 3:**
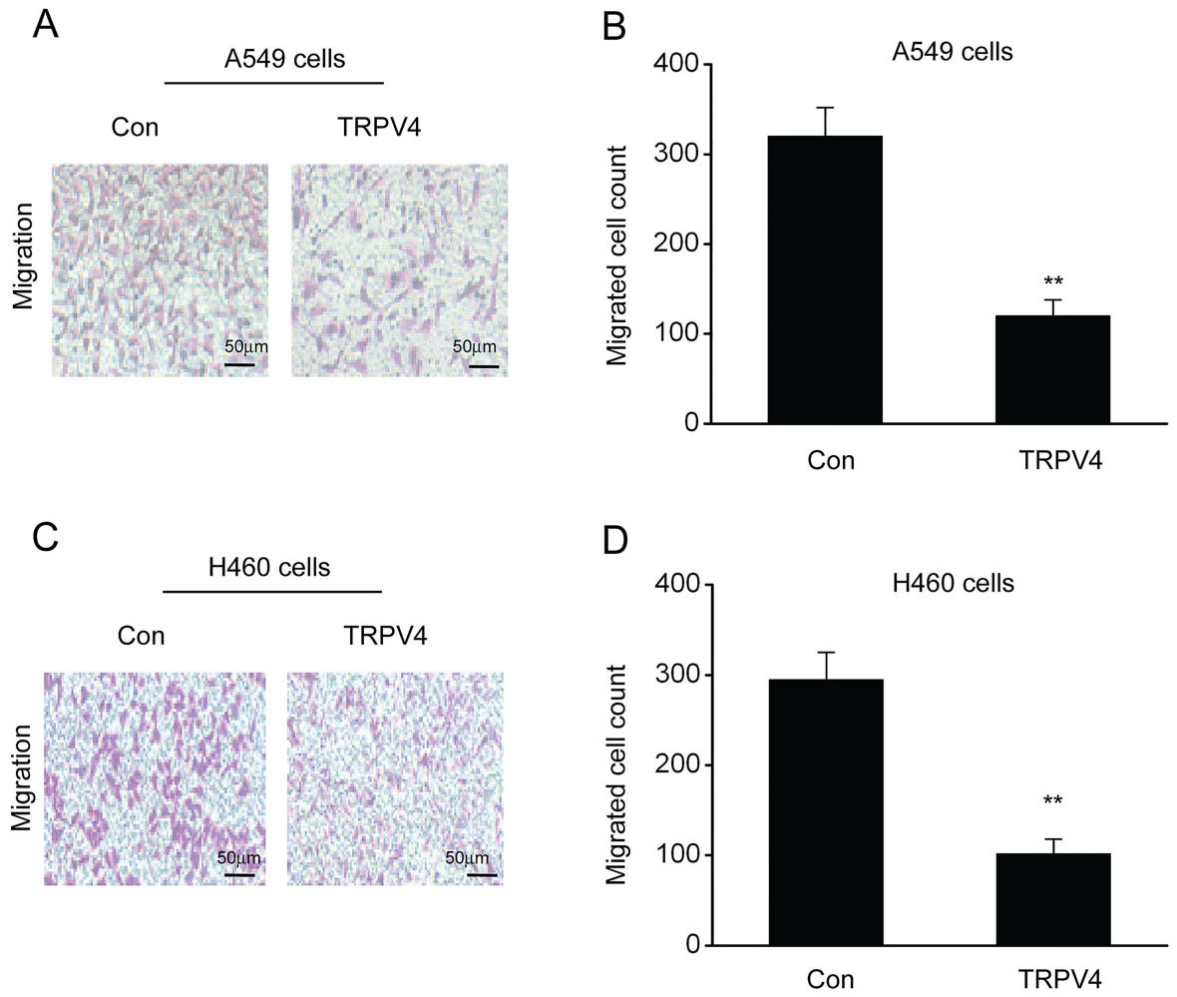
Overexpression of TRPV4 suppressed migration of lung cancer (LC) cell lines. **A**, **C**, Images showing migrated A549 (**A**) and H460 (**C**) cells on the bottom surface of transwell membranes in A549 cells transfected with the control plasmid pcDNA3.1(+) (Con) and plasmid pcDNA3(+)/TRPV4 (TRPV4) (scale bar 50 μm). Quantity of migrated A549 (**B**) and H460 (**D**) cells in 5 random microscopy fields in different groups. Data are reported as means±SE. **P<0.01 (*t*-test).

Subsequently, we explored the effect of TRPV4 on migration of the H460 and A549 cells, using transwell assays without coating. This assay showed that migration of the H460 and A549 cells were lower than that of the control cells due to overexpression of TRPV4 (Figure 3).

### Overexpression of TRPV4 promoted the death of LC cell lines

**Figure 4:**
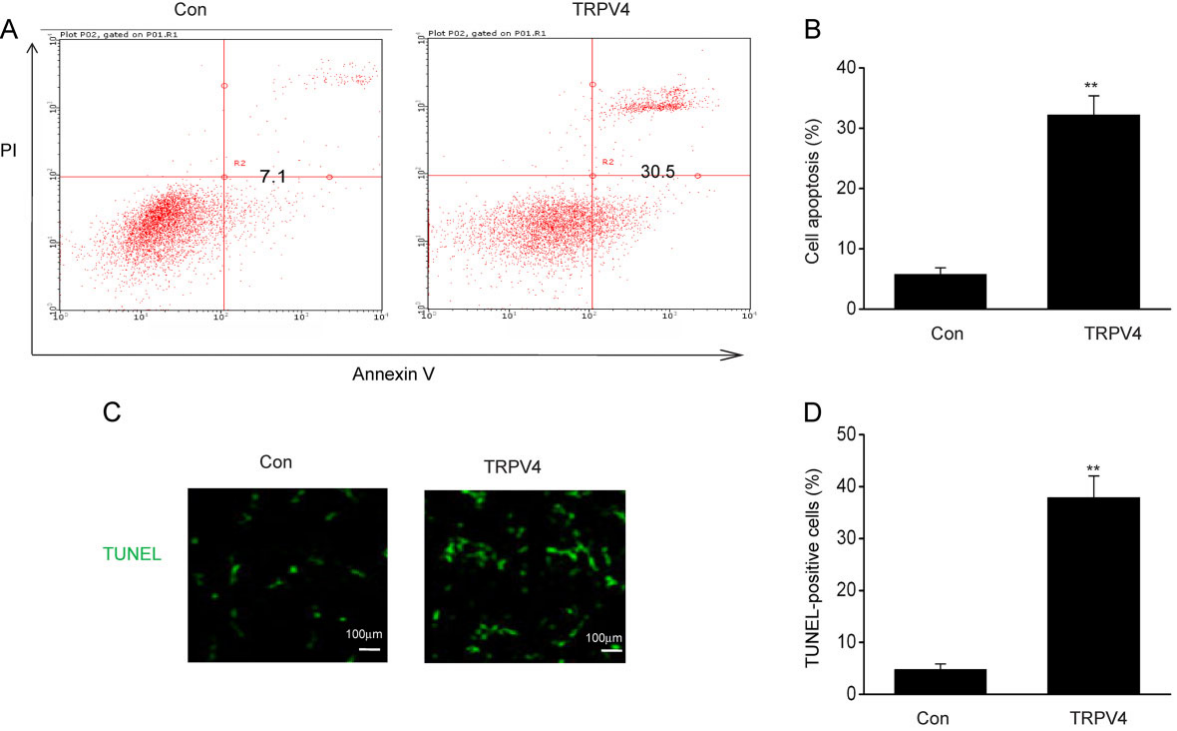
Overexpression of TRPV4 increased death of lung cancer (LC) cell lines. **A** and **B**, LC cells A549 were transfected for 24 h with the plasmid pcDNA3(+)/TRPV4 (TRPV4) or the control plasmid pcDNA3.1(+) (Con). TRPV4 promoted cell death as determined via flow cytometry. **C** and **D**, TRPV4 promoted cell death as determined via the TUNEL assay (scale bar 100 μm). Data are reported as means±SE. **P<0.01 (*t*-test).

The effect of TRPV4 on LC cell death was measured using flow cytometry of Annexin V/PI staining. The proportion of dead cells were also found to be higher in the A549 cells transfected with the TRPV4 plasmids than in the cells transfected with control plasmids (Figure 4A and B). These results were confirmed by TUNEL assays, which also showed increased cell death in the A549 cells that overexpressed TRPV4 (Figure 4C and D).

### Overexpression of TRPV4 up-regulated the expression of proteins associated with cell death in LC cell lines

**Figure 5:**
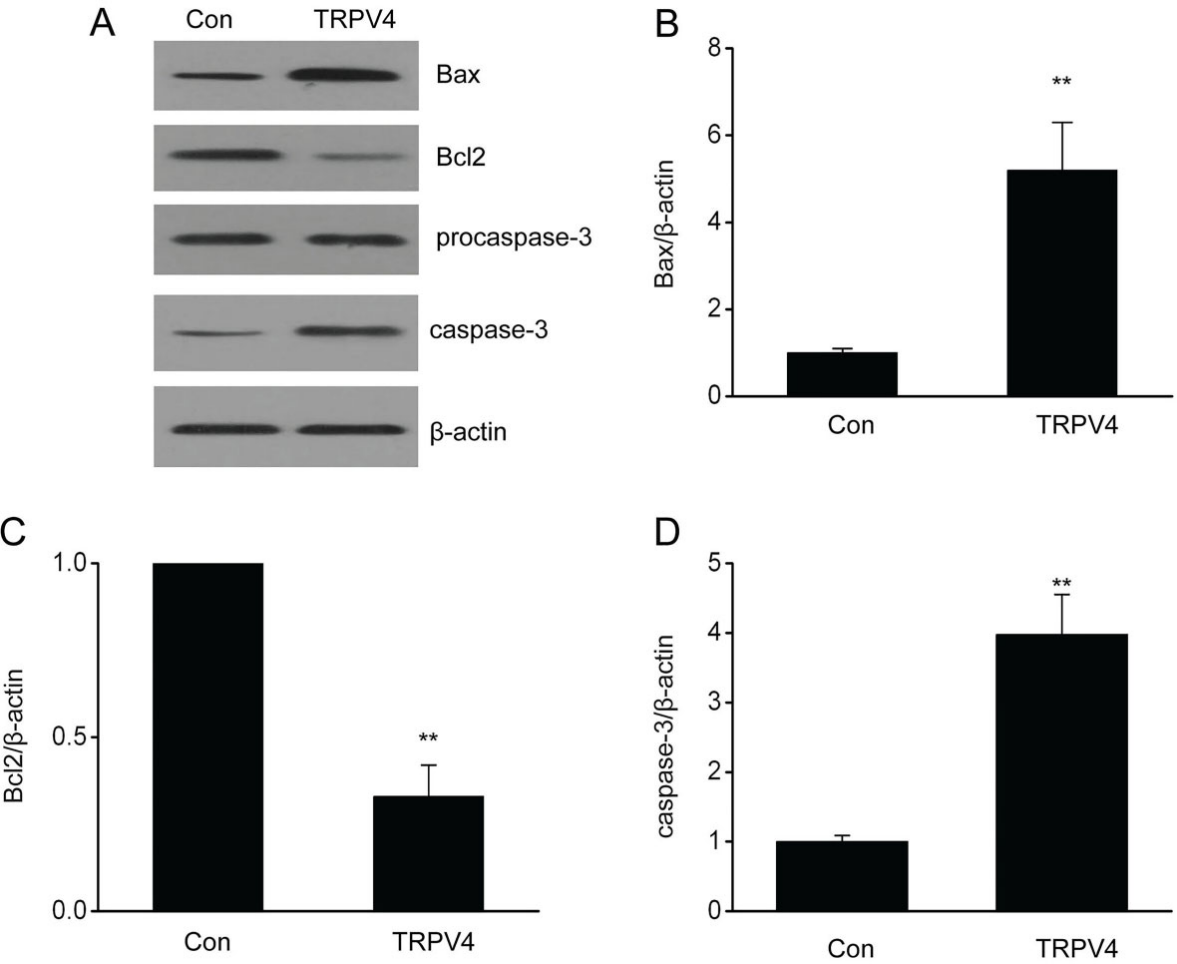
Overexpression of TRPV4 up-regulated cell death associated proteins in a lung cancer cell line. Representative immunoblots (**A**) as well as quantitative assessment of Bax (**B**), Bcl2 (**C**), and caspase-3 (**D**) in A549 cells transfected with control plasmid pcDNA3.1(+) (Con) and plasmid pcDNA3(+)/TRPV4 (TRPV4). Data are reported as means±SE. **P<0.01 (*t*-test).

Due to the effect of TRPV4 on cell death, we explored changes in the expression of proteins associated with cell death, namely Bax, cleaved caspase-3, and Bcl2 of A549 cells. Expression levels of Bax and cleaved caspase-3 were significantly higher, whereas the expression level of Bcl2 was significantly lower in the A549 cells transfected with a TRPV4 expressing plasmid than those in the control group (Figure 5). These findings showed that TRPV4 expression up-regulated proteins associated with cell death in the A549 cells.

### Overexpression of TRPV4 promoted p38 MAPK pathway stimulation in LC cell lines

**Figure 6:**
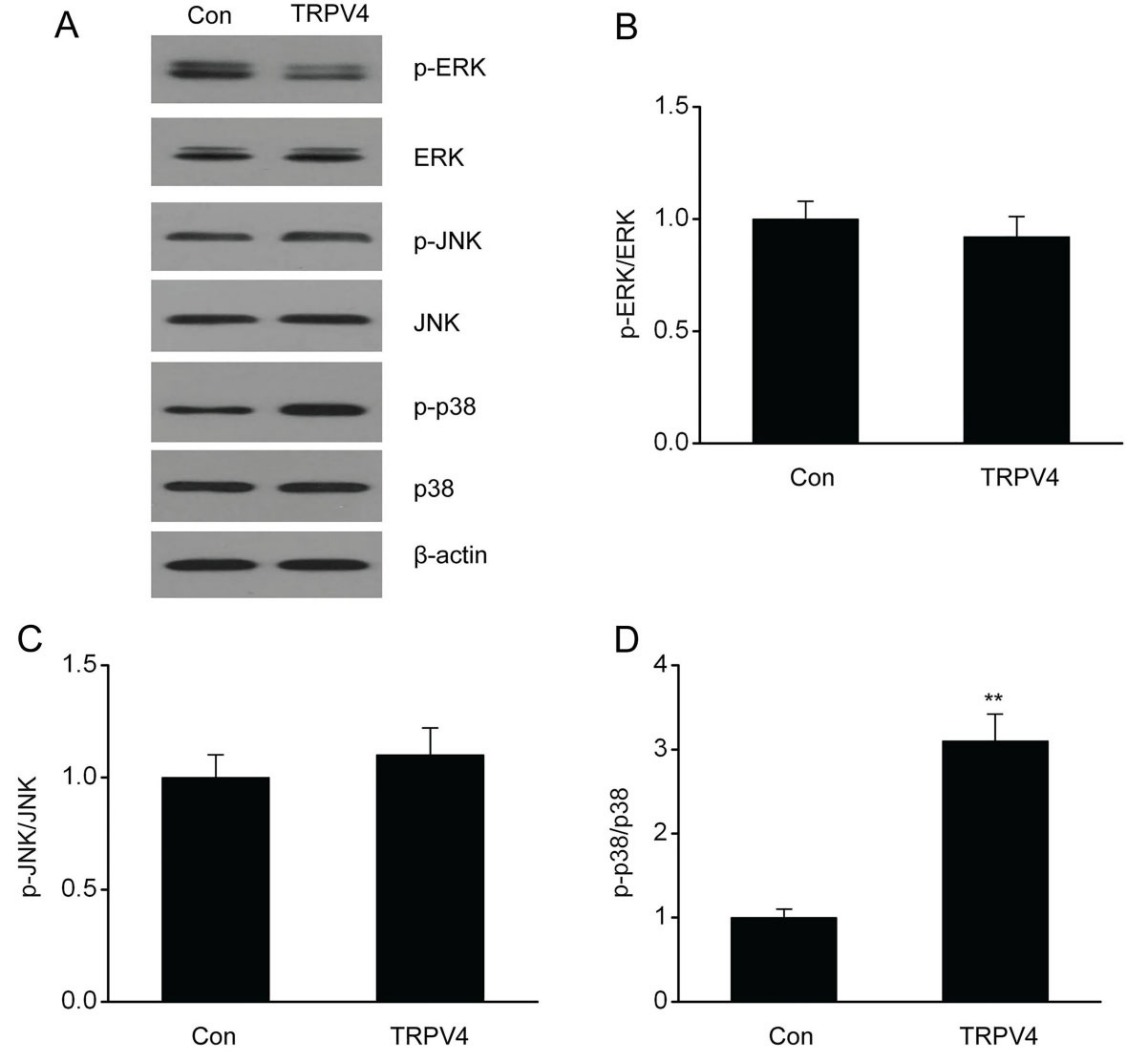
Overexpression of TRPV4 induced p38 MAPK pathway stimulation in a lung cancer cell line. Representative immunoblots (**A**) as well as quantitative assessment of p-ERK (**B**), p-JNK (**C**), and p-p38 (**D**) in A549 cells transfected with control plasmid pcDNA3.1(+) (Con) and pcDNA3(+)/TRPV4 plasmid. Data are reported as means±SE. **P<0.01 (*t*-test).

Because MAPK pathways affect the proliferation as well as the death of malignant cells (19), we explored the influence of TRPV4 on MAPK pathway stimulation. Phosphorylation of JNK and ERK was unaffected in the A549 cells transfected with TRPV4 (Figure 6). However, p38 phosphorylation was considerably higher in the A549 cells transfected with TRPV4 than in control cells, indicating that TRPV4 promoted stimulation of the p38 MAPK pathway.

### TRPV4 effect on LC cells was eliminated by blocking the p38 MAPK pathway

**Figure 7:**
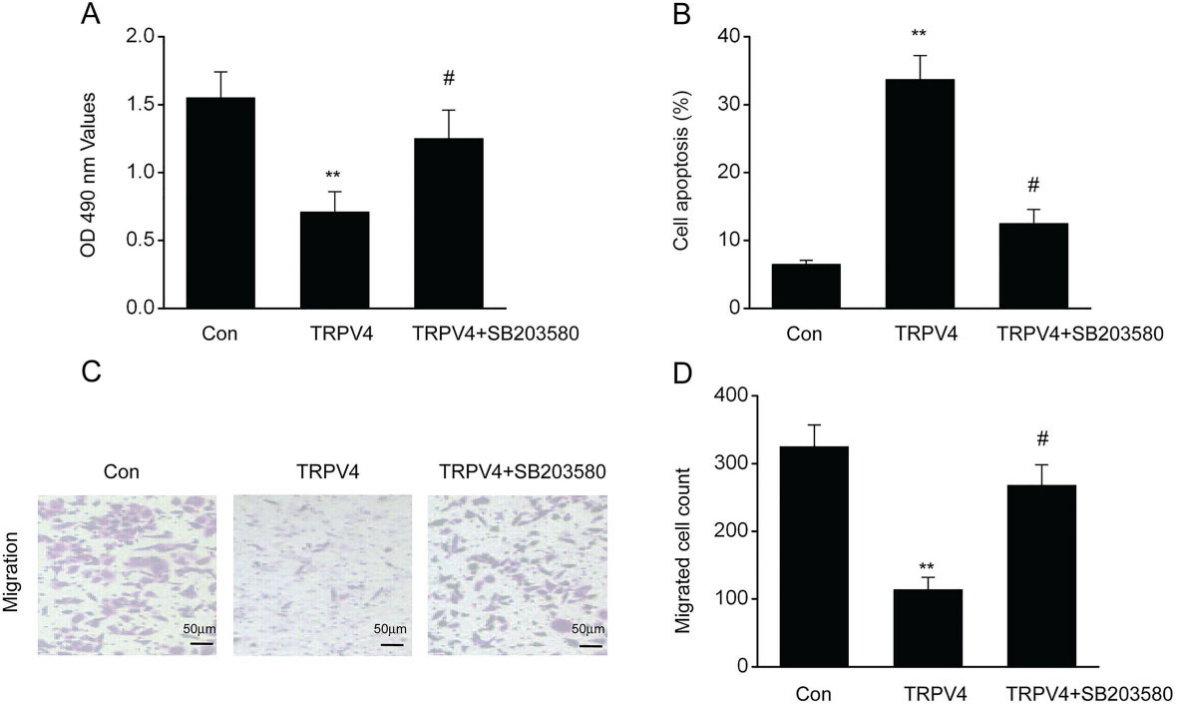
Effect of TRPV4 on lung cancer (LC) cells was eliminated by blocking the p38 MAPK pathway. LC A549 cells were supplemented with the p38 MAPK inhibitor SB203580 (10 μM) for 1 h and subsequently underwent 24 h transfection with either plasmid pcDNA3(+)/TRPV4 (TRPV4) or a control plasmid pcDNA3.1(+) (Con). **A**, MTT assay was used to assess A549 proliferation. **B**, Flow cytometry was used to assess apoptosis. **C**, Images revealing migrated A549 cells on the bottom surface of the transwell membranes (scale bar 50 μm). **D**, Quantity of migrated A549 cells in 5 random fields under the microscope. Data are reported as means±SE. **P<0.01, compared to the control group, ^#^P<0.01, compared to the TRPV4 group (ANOVA).

Our previous findings had convincingly proven that TRPV4 promoted the death of LC cells as well as the activation of p38 MAPK. To investigate whether stimulation of p38 MAPK contributed to LC cell death, we supplemented the A549 cells with SB203580, a specific inhibitor of p38 MAPK. An MTT assay proved that the presence of SB203580 eliminated the reduced proliferation and elevated cell death seen in the A549 cells transfected with a TRPV4 expressing plasmid (Figure 7A and B). Moreover, the reduction in A549 migration caused by the overexpression of TRPV4 was also eliminated by SB203580 (Figure 7C and D). These findings suggested that TRPV4 induced A549 cell death and suppressed its proliferation and migration through the p38 MAPK pathway.

## Discussion

In this study, we explored the effects of TRPV4 in human LC cell lines and observed that excessive TRPV4 promoted LC cell death through the p38 MAPK pathway. This suggestion relied on the following findings. First, our research proved that TRPV4 was down-regulated in LC tissues compared with non-malignant surrounding tissues. Second, we showed that TRPV4 induced cell death and suppressed the migration and proliferation of LC cell lines. Third, we discovered that TRPV4 enhanced stimulation of the p38 MAPK pathway, and that pharmacological inhibition of the pathway eliminated the influence of TRPV4 on LC cell lines.

LC is the most frequently seen neoplasm worldwide (20). The currently available treatments for terminal or metastasized LC have a poor prognosis for survival and serious side effects (21). Consequently, it is crucial to understand the accurate molecular mechanism of LC etiology and development as well as related signaling pathways (22). Furthermore, innovative approaches to the treatment of LC require investigation (23). Numerous studies on the TRPV4 channel function in various normal tissues and cells, such as epithelial cells, skin, and hypothalamic 4B cells, have been conducted (24-27). Nevertheless, little research has been done on the effect of TRPV4 channels on the generation and development of malignancies. Huang et al. (28) proved that overnight GSK101 exposure had no effect on HaCaT cell survival; however, Olivan-Viguera et al. (18) demonstrated that TRPV4 stimulated via GSK101 was able to inhibit the proliferation of melanoma cells and HaCaT keratinocytes. Lee et al. (29) discovered that TRPV4 suppressors are able to efficiently prevent the invasion as well as the migration of human breast cancer cells 4T07 that displayed TRPV4 up-regulation. Nevertheless, the current understanding of the expression as well as the activity of TRPV4 in LC cells is insufficient. Our research revealed that TRPV4 expression is down-regulated in LC tissues compared with the non-malignant surrounding tissues. Furthermore, our results showed that over-expression of TRPV4 induced cell death and suppressed the migration and proliferation of LC cell lines. These findings indicated that TRPV4 could serve as a promising target in the treatment of LC.

MAPKs modulate various cellular reactions caused by extracellular stimuli (30-32). The p38 MAP kinases, one of the major subgroups, participate in various complicated biological reactions, including proliferation, apoptosis, differentiation, migration, and invasion (33). Malfunction of p38 MAPK in patients is associated with decreased survival in patients suffering from malignancies (e.g., breast, bladder, hepatic, prostate, and LC) (34,35). p38 MAPK has a versatile role during apoptosis regulation, varying based on the type of stimulus in a cell type-specific manner (36). Apart from regulating viability, the crucial effect of p38 MAPK on invasion as well as migration provides important reasons to target the pathway to inhibit malignancy metastasis (33). This particular activity of p38 MAPK seems to rely on both the cell type and the stimuli and/or isoform that is being stimulated. In this study, we proved that overexpression of TRPV4 selectively promoted stimulation of p38 MAPK whereas it had no effect on levels of JNK or ERK phosphorylation. Furthermore, pharmacological inhibition of the p38 MAPK pathway eliminated the effects of TRPV4 on LC cell lines.

Briefly, the findings of this research showed that TRPV4 promoted cell death and inhibited migration as well as proliferation via the p38 MAPK pathway in human LC cell lines, indicating that TRPV4 could serve as a promising innovative target for the treatment of LC in the future.
